# Surface Electromyography: What Limits Its Use in Exercise and Sport Physiology?

**DOI:** 10.3389/fneur.2020.578504

**Published:** 2020-11-06

**Authors:** Francesco Felici, Alessandro Del Vecchio

**Affiliations:** ^1^Department Motor, Human and Health Sciences, Rome University Foro Italico, Rome, Italy; ^2^Department Artificial Intelligence in Biomedical Engineering, Friedrich-Alexander University, Erlangen, Germany

**Keywords:** HDsEMG, sport, exercise physiology, biomechanics, EMG, motor unit

## Abstract

The aim of the present paper is to examine to what extent the application of surface electromyography (sEMG) in the field of exercise and, more in general, of human movement, is adopted by professionals on a regular basis. For this purpose, a brief history of the recent developments of modern sEMG techniques will be assessed and evaluated for a potential use in exercise physiology and clinical biomechanics. The idea is to understand what are the limitations that impede the translation of sEMG to applied fields such as exercise physiology. A cost/benefits evaluation will be drawn in order to understand possible causes that prevents sEMG from being routinely adopted. Among the possible causative factors, educational, economic and technical issues will be considered. Possible corrective interventions will be proposed. We will also give an overview of the parameters that can be extracted from the decomposition of the sHDEMG signals and how this can be related by professionals for assessing the health and disease of the neuromuscular system. We discuss how the decomposition of surface EMG signals might be adopted as a new non-invasive tool for assessing the status of the neuromuscular system. Recent evidences show that is possible to monitor the changes in neuromuscular function after training of longitudinally tracked populations of motoneurons, predict the maximal rate of force development by an individual via motoneuron interfacing, and identify possible causal relations between aging and the decrease in motor performance. These technologies will guide our understanding of motor control and provide a new window for the investigation of the underlying physiological processes determining force control, which is essential for the sport and exercise physiologist. We will also illustrate the challenges related to extraction of neuromuscular parameters from global EMG analysis (i.e., root-mean-square, and other global EMG metrics) and when the decomposition is needed. We posit that the main limitation in the application of sEMG techniques to the applied field is associated to problems in education and teaching, and that most of the novel technologies are not open source.

## Introduction

Traditionally, the acquisition of EMG signals is prevalent to clinical contexts—neurology, orthopedics, physiatry—and, to the best of our knowledge, it almost always involves needle/fine wire EMG. The usage of surface EMG (sEMG) for the study of motor control is primarily applied to research environments. Only few clinical laboratories adopt sEMG measures to estimate the health and potential neuromuscular changes of the nervous system. The limited usage of sEMG only to research environments is mainly dictated by the fact that the global EMG signals is associated to the activity of many motor units and the properties of the tissue between the electrodes and the muscle fibers [i.e., the volume conductor, for (1–7)]. However, the parallel development of high-density EMG electrodes (grids of more than >32 electrodes with low interelectrode 5–10 mm spacing) with blind source separation algorithms allows. for the first time, the identification of individual motor unit spike trains (8, 9), which have been validated using both simulations and intramuscular EMG recordings (10, 11). The access to a representative population of motor neurons allows the identification of the responsible mechanisms for the development of muscle force (12).

Despite these advantages, there are major limitations for the access of sEMG analysis to exercise physiology. These limitations include the education of the exercise and sport physiologist, occupational therapists, and fitness and training experts. There is now sufficient evidence to show that it is possible to interface the output of the human spinal cord by applying high-density grids of electrodes on the surface of the muscles (8, 13–15). This type of technology allows to study the behavior of a representative populations of spinal motor units in an unprecedented way and in a fully non-invasive manner. This is obviously of utmost importance for the above listed professional, including those working in the field of preventive and adapted physical activity.

Nevertheless, as already pointed out by many authors (1, 2, 4, 12, 16, 17) global sEMG analysis shows important limitations that impede the access to the neural drive (ensemble of motor unit action potentials) to the muscle. The latter plays a significant restriction against the widespread use, acceptance, and relevance of sEMG data analysis from both researchers and professionals. Due to the fact that it is so deceptively easy to collect reasonable sEMG data, researchers tend to underestimate this technique, while movement professionals tend to overestimate it. There seems to be a failure to communicate among sEMG experts and potential users. In fact, if we look at citations our recent and less recent papers are receiving, it is immediately apparent that ~90% comes from colleagues directly involved in the same field (Scopus source). The lack of citation from clinical and applied journals is, overall, associated to communications challenges between the clinical, professional and research fields. However, there are no doubts that the limitations in the accessibility and teaching of novel technologies (both at the software and hardware level) limits the diffusion of sEMG technique and data analysis outside the laboratories.

**Figure 1:**
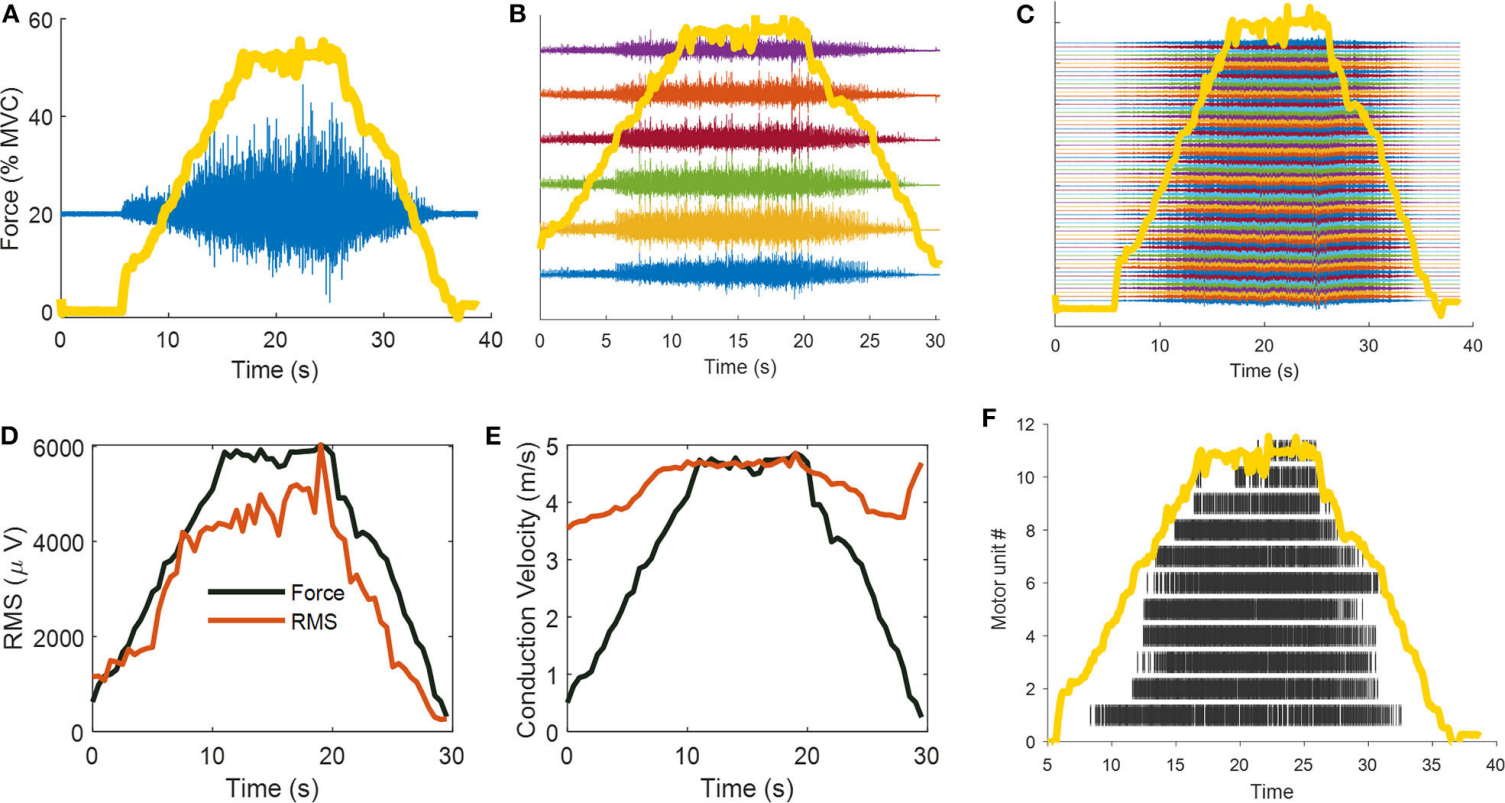
Classic surface EMG recording frameworks: the evolution from bipolar, linear arrays, and high-density EMG configurations. In this example, a 26 year-old men performed isometric linearly increasing ramp contractions up to 50% of maximal voluntary force. **(A)** Bipolar (double differential, in blue) surface EMG recordings superimposed on ankle-dorsiflexion force (yellow line). **(B)** Six double-differential EMG recordings. **(C)** Sixty-four monopolar EMG signals. **(D)** The most common parameter that is extracted from a bipolar EMG signals is its amplitude. The surface EMG amplitude is weakly associated to the effective neural drive to the muscles due to the effect of volume conductor, amplitude cancellation, and random positions of motor units in the muscle tissue. The strong correlation with joint force, as shown in **(D)**, can be misinterpreted as the effective neural output that reach the muscle per unit time. This speculation is however wrong, since the amplitude of the surface EMG can only increase monotonically with force as it corresponds to an approximate sum of the number of motor unit action potential that reach the muscle per unit time (therefore both motor unit recruitment and motor unit discharge rate), and both of these measure increase with force (note that motor unit recruitment reaches a different plateau for different muscles, and is in the range 40–95% of maximal voluntary force). However, the influence of volume conductor, amplitude cancellation and divergent associations of EMG amplitude with motor unit action potential amplitude within different muscles, impedes the usage of EMG amplitude as a biomarker of neural function. Indeed, the shape of the action potential of the motor unit is only determined by the muscular properties. For some limited prosthetic applications, the amplitude of the EMG may be used as a proxy of the neural commands, as shown in **(D)** (the coefficient of correlations and Pearson *P*-value between force and RMS in **(D)** are, respectively, *R* = 0.95, and *P* = 8.39e-31). **(E)** Muscle fiber conduction velocity (MFCV) as estimated from 6 bipolar EMG signals. Muscle fiber conduction velocity is a basic physiological parameter related to the diameter of muscle fiber, pH level, and ion concentration. MFCV increases linearly with force with similar correlation to RMS, however, it can be only estimated if the muscle fiber are disposed in parallel with the recording electrodes. Note that the conduction velocity is sensitive to the activity of motor unit action potentials and is positively associated to motor unit conduction velocity and recruitment thresholds. The high-conduction velocity at the end of the ramp contractions in **(E)** is due to the non-propagating components (intrinsic noise of the EMG). Indeed, the coefficient of correlation between channels at this time point was very low *R* = 0.23, as opposed to the ramp contractions with high EMG activity (average correlation between channels *R* = 0.84 ± 0.018). **(F)** The high-density EMG were decomposed with blind source separation approaches, identifying the unique spatiotemporal representation that constitute the original signal (i.e., the motor unit action potentials). The raster plot shows 11 motor units during that were identified during the contraction. Note the progressive recruitment of the motor units.

Figure 1 shows an illustration of the limitations and potentials of surface EMG analysis, from classic bipolar EMG recordings (global EMG estimates) and the information that can be extracted through the decomposition of the EMG signals.

The motor unit, which comprises an alpha motoneuron and the muscle fibers innervated by its axon, represents the output final common pathway of the central nervous system. Therefore, the behavior of these individual neural cells, provides important information on the status and health of the neuromuscular system. Indeed, motoneurons convey information from afferent and efferent fibers within the sensorimotor system and generate force by sending action potentials to group of muscle fibers (the muscle units). Any potential changes in the distribution and strength of common input sent to motoneurons can likely be assessed by decoding a representative population of motoneurons, as we showed in many studies (15, 18–21).

Our group has recently provided evidence that is possible to predict important behavioral parameters such as the maximal contractile speed of muscle (20, 22) and the neural changes following training (21) by identifying the discharge timings of the motoneurons using high density sEMG recordings (HDsEMG). Using this technique, we showed for the first time that the discharge characteristics of motor units in the tibialis anterior muscle tracked across the intervention change after 4 weeks of strength training consisting of isometric voluntary contractions. These adaptations included increase in motor unit discharge rate, decreases in the recruitment-threshold force of motor units and a similar input–output gain of the motor neurons during submaximal contractions at the same relative maximal force level (before and after training). The findings indicate that the adaptations in motor unit function may be attributable to changes in synaptic input to the motor neuron pool or to adaptations in intrinsic motor neuron properties. Most importantly, they point out that is possible to associate a large portion of the spinal cord output to function (e.g., the changes in force with training are due to neural mechanisms).

Another critical aspect that is fundamental for sport and preventions of injuries is the rate of force development of a muscle (23–26). With regard to this issue, we showed that the rate of force development is significantly correlated with the very early phase of the neural drive, which takes place even before the onset of movement and that can be characterized in terms of discharge rate and motor unit recruitment speed (20). This characteristic implies that the interfacing with the spinal cord allows accurate prediction of the contractile speed. Therefore, monitoring these physiological parameters is likely necessary in order to train and rehabilitate the neuromuscular apparatus. Moreover, the fluctuations in joint force during isometric steady state contractions can be predicted by low-pass filtering of the neural drive to the muscle (27), which implies functional associations between the common motoneuronal oscillations and force tracking accuracy. This is particularly important for the aging neuromuscular system, which shows poorer performances in force accuracies. Indeed, there are correlations between the variability in the output of the common motoneuronal fluctuations and force accuracy (28, 29).

## Translation of SEMG from Basic Research to Applied Environments

We investigated the role of education by looking at the first 100 bachelor and master courses, of the top 100 university (extracted via QS World Ranking and Shanghai Ranking) in the area of Human Movement, Biomechanics, Sport Science, Physiotherapy and Exercise Physiology, and we found that only a very limited number (5% of the total) teaches students the fundamental principles for studying the neural control of muscles at the direct motor unit level. Specifically, we aimed at finding direct evidence of theoretical or practical (lab-based) teaching of the fundamental principles of motor unit physiology and the potential methods available to the research/clinical environments. However, the full details of the course of neurophysiology for several university (~24% of total) was not publicly available or not found.

One major limitation is the access to these technology to both research and non-research environments. There is critical need to open the access of these technologies and to instruct teachers across the disciplines of these fundamentals. Although the physiological principles and engineering developments strongly suggest that it is now the time to routinely monitor the spinal cord output by non-invasive high-density EMG recordings, we are failing to generalize and give access of these developments to the current generation of students, which would improve the applied translation in order to potentially predict and cure pathologies of the neuromuscular function. This has an impact, for example, on the student awareness level of some very simple points related to the correct collection of sEMG data. Issues such as electrodes location, innervation zone detection, skin preparation, movement artifacts and sweating are very often ignored.

Before coming to the most up to date sEMG technical developments, let us consider the simplest case, were one wants to describe the timing of muscle activations during the execution of a given motor task—as walking on a flat terrain—by means of bipolar sEMG recordings. Human locomotion, in its various forms, can be used as a paradigmatic example of application of sEMG to the study of dynamic exercise. Walking involves a series of coordinated movements of the body segments, implying an interplay of muscular forces and external forces (inertial, gravitational, and reaction forces) in order to achieve locomotion of the body (30). The importance of having a complete and precise description of human walking is evident: this knowledge provided significant contributions in various fields: from rehabilitation to exercise science. However, gait analysis data, to be used at their best, should be organized according to some standards.

As already pointed out by previous papers, to facilitate the systematic interpretation of sEMG gait data, stride-to-stride variability needs to be assessed before any particular stride is considered representative of subject's performance (31). Averaging multiple data will provide linear envelopes or ensemble averages of sEMG data that can then be used to identify gait deviation or changes intervening because of fatigue, a change in speed of progression or walking style [from race walking to stroke patients (32)]. It must be stressed once again that sEMG data alone are not enough, in the majority of cases, to obtain a complete and meaningful picture. Pertinent temporal parameters that should be included are walking velocity, cadence, stride time, step and stride length and duration, and double support and single support intervals.

Moreover, it would be important to associate gait parameters to the firing synchrony of multiple motoneuron pools. For example, the neural motor commands extracted from factorization analyses applied to multi-EMG recordings (33) may be analyzed at the direct motoneuronal level, by performing correlation and classification analyses to identify the unique spatiotemporal patterns (sequence of different population of motoneurons discharge timings) that are responsible for specific patterned behavior such as gait. Moreover, the identification of populations of motor neurons innervating different muscles would also allow to perform synchronization analyses revealing associations between specific motor nuclei and gait cycles. All this information cannot be extracted from global EMG estimates (i.e., EMG amplitude or conduction velocity).

It is immediately evident from the above example that an interdisciplinary approach is needed, commonly known with the term neuromechanics (34). The many interests involved produced a variety of competences and applications, but, at least in Italy, there are only scanty traces of any basic course of physics, mathematics, neurobiological data processing included in the curricula of physiatry, orthopedics, physiotherapists, and exercise/sport physiologists, which represents the potential professional users of this technique.

As described above, it is clear that to have an overview of our body in motion we must link direct cellular behavior to function. These tasks can now be achieved by the decomposition (with the use of blind source separation algorithms) of HDsEMG signals. Although the acquisition and analysis is relatively automatic, there is need of careful inspection of the signals (35). There is a large number of parameters that can be extracted from the discharge of populations of motor units and each of these parameters has a specific neurophysiological determinant. In a recent Tutorial article (35), we described the physiology and applications of these parameters. It is clear that these techniques are still relatively novel, therefore time is needed to train and grant access to everyone and assess its potential clinical utility. Specialized courses across the universities and clinical environments are needed in order to train and teach the future generation of sport and exercise physiologists, physiotherapy and clinical neurophysiology.

Another important main factor limiting the wide use of these technique is represented by the limited access to the software needed for separating the motor unit action potentials in the sEMG. Different research groups have proposed algorithms to decompose the sEMG interference signals, however, none of these approaches have been published open source. All of these problems impede the translation of HDEMG analysis to the applied field.

## Costs/Benefits

The aim of this short communication also includes to provide an opinion on the costs/benefits ratio regarding the use of novel sEMG techniques. First, what is meant, or better felt, by academics with respect to benefits. The probability of a work to be published is surely one of the perceived benefits. More important than this, though, is the probability to be cited a significant number of times. This, in turn, has an impact on the probability of a successful grant application. In the case of sEMG based papers this probability is limited due to the relatively small number of researchers in the field. Besides, also in sEMG parochial environment, the most cited papers are, or at least were until recently, those more technical in nature, while basic and applied physiology papers were less cited and appeared on medium level journals. As a consequence, in this specific application, basic and applied physiology have generated limited benefits to academics, both in terms of their personal achievement and in terms of financial support. It seems that this trend is now quickly (1, 20, 29) changing due to the uncontroversial advancement in sEMG technique of the last 10 years coupled to the increased power of the analytical tool specifically designed to decipher the sEMG signal (8). On the other hand, the lack of a widespread diffusion of a correct information on sEMG among professionals is still a serious drawback and this continues to prevent professionals from the routine adoption of sEMG in their evaluation protocols.

It is also to be considered that professionals need to have sEMG equipment that are feasible for the field usage. To this respect, wearable devices although promising are not fully mature, particularly on a high-density EMG scale (36).

What about benefits for companies? Selling devices to a large set of potential users is the obvious goal of a firm. In spite of this, the dissemination of information about their specific products is left mostly to standard internet channels. In our opinion, companies should increase their investments on peer to peer formation of end users provided these latter have been adequately formed by academics (see above).

Companies should also think in terms of application to sport, rehabilitation, clinical evaluation in order to increase the demand from the market. This will increase their earnings, allowing at the same time a reduction of the monetary costs for the clients, irrespective from being an academic or a professional. As a matter of fact, not only the most sophisticated and reliable equipment are somehow still very expensive (>15,000 euros for a plug-in hardware and software devices with ~ 200 EMG channels), but so are the consumable associated with these instruments.

At the very end, it turns out that to overcome the present limitations of a large diffusion of sEMG in applied and allied sciences, a joint serious effort is needed from the many actors involved. There is not a single responsible for this situation and this, maybe, makes the problem complicated.

## Author Contributions

All authors contributed in equal part to conceptualization and writing of the manuscript.

## Conflict of Interest

The authors declare that the research was conducted in the absence of any commercial or financial relationships that could be construed as a potential conflict of interest.
